# Bactericidal and plant defense elicitation activities of Eucalyptus oil decrease the severity of infections by *Xylella fastidiosa* on almond plants

**DOI:** 10.3389/fpls.2023.1122218

**Published:** 2023-03-15

**Authors:** Laura Montesinos, Aina Baró, Beatriz Gascón, Emilio Montesinos

**Affiliations:** Institute of Food and Agricultural Technology-CIDSAV-XaRTA, University of Girona, Girona, Spain

**Keywords:** eucalyptus oil, bactericidal, plant defense elicitor, plant infections, *Erwinia amylovora*, *Xylella fastidiosa*

## Abstract

The activity of Eucalyptus essential oil against eleven strains pertaining to six species of plant pathogenic bacteria was studied using growth inhibition and contact assays. All strains were susceptible to the formulation EGL2, and *Xylella fastidiosa* subspecies and *Xanthomonas fragariae* were the most sensitive. The bactericidal effect was strong causing 4.5 to 6.0 log reductions in survival in 30 min at concentrations in the range of 0.75 to 15.0 μl/ml depending on the bacteria tested. Transmission electron microscopy of the formulation EGL2 against the three *X. fastidiosa* subspecies studied allowed the observation of a strong lytic effect on bacterial cells. In addition, the preventive spray application of EGL2 to potted pear plants subsequently inoculated with *Erwinia amylovora* significantly decreased the severity of infections. Almond plants treated by endotherapy or soil drenching, and then inoculated with *X. fastidiosa* showed a significant decrease in disease severity as well as in the levels of the pathogen, depending on the strategy used (endotherapy/soil drenching, preventive/curative). The treatment by endotherapy in almond plants induced the expression of several genes involved in plant defense. It was concluded that the reduction of infections by the Eucalyptus oil treatments was due to the combination of its bactericidal and plant defense induction activities.

## 1 Introduction

Worldwide losses in crop yields due to diseases, and their implications for global food demands, create the need for improving disease management. IPPC Secretariat (2021a) estimated annual loses up to 40% of global crop production caused by pests, where plant diseases represent a global economy cost over $220 billion, and international standards for phytosanitary measures include protecting sustainable agriculture and improving global food security, as well as protecting the environment (IPPC Secretariat, 2021b). European Commission aim to reduce by 50% the currently homologated chemical pesticides by 2030 (EC, Plant protection in EU agriculture 2022), but in fact, control of most bacterial diseases is only reasonably accomplished by using copper compounds. However, these compounds are not sufficiently effective in most devastating diseases such as those caused by *Pseudomonas syringae* pv. syringae (bacterial canker of pear), *P. syringae* pv. actinidiae (bacterial canker of kiwi fruit), *Xanthomonas arboricola* pv. pruni (bacterial spot of almond) or *Erwinia amylovora* (fire blight of pear, apple) (Iacobellis et al., 2005; Vanneste et al., 2008; Palacio-Bielsa et al., 2010).

Even more difficult is managing diseases caused by the fastidious phytopathogenic bacteria *Xylella fastidiosa* or *Candidatus* Liberibacter (Blaustein et al., 2019; Moll et al., 2022). *X. fastidiosa* is responsible for emerging diseases in Europe affecting mainly olive and almond, and there are still no effective methods to cure infected plants, due to the lack of effective bactericides and the difficulty to access to the vascular system, where the pathogen establishes. This pathogen is exclusively transmitted by xylem fluid-feeding insects, by grafting or budding and can infect more than 600 plant species (EFSA, 2022). This wide spectrum of hosts combined with genetic plasticity, favors the spread of the pathogen, and make its control extremely difficult (Baldi and La Porta, 2017; Denancé et al., 2019; EFSA, 2021). Since the first detection of X. fastidiosa subsp. pauca in olive plants in Apulia region, Italy in 2013, about 5 million trees has been infected or dead, representing losses of about 10% of Italian olive oil (White et al., 2020). In Spain, *X. fastidiosa* was first detected in Balearic Islands in 2016 and to date, more than 80% of almond trees are affected by almond leaf scorch disease (ALS) (Morelli et al., 2021; Olmo et al., 2021). Similarly, in 2017, *X. fastidiosa* subsp. *multiplex* was detected in diseased almond trees in the Valencian Community (Spain), and in 2021, the infected area reached 2292 ha, and more than 100,000 diseased almond trees have been destroyed (Morelli et al., 2021). Several strategies have been assessed to control *X. fastidiosa* diseases on plant hosts, including chemical control using oxytetracycline or Zn/Cu citric biocomplex foliar treatments (Amanifar et al., 2016; Dongiovanni et al., 2017; Scortichini et al., 2018; Bruno et al., 2021), stimulators of plant defense responses (Zhang et al., 2019; Moll et al., 2022), biological control using antagonistic endophytes (Baccari et al., 2019) or different agricultural practices. Some of these treatments can reduce symptoms, and in some few cases, even decrease the population levels in infected plants or trees. However, the main concern is that no cure has been found to be effective in the control of *X. fastidiosa* in infected trees.

Thus, there is a strong need for new products for bacterial disease control, and specially for the emerging and re-emerging diseases of tree crops (Sundin et al., 2016; Sharma et al., 2022). In recent years, the development of biopesticides and particularly non-microbial biopesticides based on natural products are experiencing an increasing growth, as they fulfill the regulations associated to plant protection, such as sustainability, biodegradability and minimal toxicity to humans and environment (Leahy et al., 2014). Many plant extracts or essential oils (EOs) appear as alternative control strategies, since their efficacy against a wide range of pathogens and pests has been confirmed *in vitro* and to a lesser extent, *in planta* (Raveau et al., 2020). Particularly, different studies reported *in vitro* antibacterial activity of EO such as EO from *Satureja hortensis* L., *Cleistocalyx peraculatus* or *Eucalyptus globulus* against *E. amylovora*, *Xanthomonas campestris* pv. vesicatoria or *X. fastidiosa* (Bajpai et al., 2010; Karami-Osboo et al., 2010; Brentini et al., 2018).

In addition, it is important to point out that the regulatory process for registration of bioactive natural products as pesticides may be faster than for conventional chemical products (Mohan et al., 2011).

Different EO-based products have been developed and screened for their fungicidal, herbicidal, and insecticidal activity or as growth regulators, and an increasing number of these products have been homologated for their use in agriculture and commercialized (Raveau et al., 2020; Pathma et al., 2021). Among commercially available botanical derived-biopesticides, there are some described as antimicrobial agents and/or inducers of plant defense, and are effective in controlling bacterial diseases in several crops such as tomato or wheat. In this context, tea tree oil has been reported to exhibit antifungal and antibacterial properties, inhibiting powdery mildew of barley (Terzi et al., 2007). Also, a salicylic acid (SA) analog or ethanolic extracts of Giant Knotweed can induce resistance in a wide range of hosts (Ramasamy et al., 2015; Margaritopoulou et al., 2020). However, there is a lack of EO-products against plant pathogenic bacteria and bacterial diseases. Eucalyptus EO was shown to have antibacterial activity (Tan et al., 2008; Bachir and Benali, 2012), and presents a low risk profile, as it is commercially available for human health and as flavoring agents in food (Batish et al., 2008). Interestingly, neither Eucalyptus EO nor active substance derived from it are approved for use as biopesticides, neither in Europe nor in the United States (https://food.ec.europa.eu; https://epa.gov) .

The aim of the present work was to evaluate the effect of a Eucalyptus EO (EGL2) in controlling *X. fastidiosa* infections in young potted almond plants as well as *E. amylovora* infections in pear plants. More specifically the objectives were to: (1) evaluate the antimicrobial activity of EGL2 against different plant pathogenic bacteria of economic importance, (2) assess the effect of spray treatment with EGL2 in controlling *E. amylovora* infections, (3) study the effect of different treatments with EGL2 in the reduction of ALS symptoms and levels of *X. fastidiosa* in almond plants, and (4), evaluate the effect of EGL2 treatment in the induction of defense responses in almond plants.

## 2 Materials and methods

### 2.1 Bacterial strains and growth conditions

The bacterial pathogens and growth conditions used in the study are listed in **Supplementary Table 1**. Bacterial strains were cultured at 28°C for 24 h-48 h (for *X. fastidiosa* strains, 7-10 days) and scrapped from surface to prepare suspensions adjusted to 10^8^ CFU/ml.

### 2.2 Eucalyptus oil formulation

EGL2 Eucalyptus EO was obtained from leaves and branches of *Eucalyptus globulus* plants at harvest time in winter, from October to February. Qualitative and quantitative analyses were performed using gas chromatography coupled with a flame ionization detector (GC-FID). The main components identified were 1,8-cineole (>70), limonene (<10%) and α-Terpineol (<10%) (**Supplementary Table 2**). Dilutions of the product were made in double distilled water to obtain the desired final concentrations.

### 2.3 Antimicrobial activity

Agar incorporation test method was used for growth inhibition assays for EGL2 product. Briefly, the required agar growth medium was mixed with the corresponding product concentration (5, 10, 30, 60 and 120 µl/ml), and the 10 µl of the test plant pathogenic bacteria (at final concentration of 10^8^ CFU/ml) was added at the center of the agar plate. Three replicates for product concentration were used. Positive controls containing water instead of product, and negative controls containing a bactericidal reference product were included (streptomycin and ampicillin at 100 mg/l). Plates were incubated at 28°C for 24-48 h or 5-10 days, depending on the plant pathogenic bacteria (**Supplementary Table 1**). Microbial growth was determined qualitatively, by determining the presence or absence of bacterial growth. The minimal inhibitory concentration (MIC) value was taken as the lowest product concentration with no growth at the end of the experiment.

### 2.4 Bactericidal activity

Bactericidal activity of EGL2 was determined by a contact test or *killing assay*, consisting of the exposure of the target microorganism (selected from) to an antimicrobial compound for a given time and determining the surviving cells (Montesinos et al., 2021). 100 µl of the corresponding product concentration were mixed in a microtiter plate with 100 µl of bacterial suspension (at final concentration of 5x10^7^ CFU/ml), to a total volume of 200 µl. Three replicates for each concentration and pathogen were used. Controls containing water instead of EGL2 (negative control) or a bactericidal reference product (positive control) were included. Microplates were incubated at 28°C for 30 and 120 minutes under constant shaking. The plate counting method was used to quantify the culturable cells and to assess the bactericidal activity of EGL2. Decimal dilutions of each EGL2 concentration were prepared and plated (20 µl) onto the surface of agar plates and colony forming units (CFU) were quantified at 24-48 h or 5-10 days after the incubation at 28°C.

### 2.5 Transmission electron microscopy

Alterations in bacterial membrane morphology and integrity of *X. fastidiosa* subsp. *fastidiosa*, *X. fastidiosa* subsp. *multiplex* and *X. fastidiosa* subsp. *pauca* cells after EGL2 treatment was observed by transmission electron microscope (TEM). Cells were exposed to EGL2 (6 µl/ml) for 120 min and harvest by centrifugation at 10,000 g for 10 min. The Microscopy Unit (Research Technical Services) of the University of Girona fixed pelleted cells, included in epoxy resin, and prepared the ultra-thin sections of 60 to 80 nm as described in Baró et al., 2020a. After contrasting sections with uranium acetate 2%, the samples were observed with a JEOL JEEM1400 transmission electron microscope at the Microscopy Unit of the Autonomous University of Barcelona.

### 2.6 Effect of EGL2 treatment on bacterial infections in plants

The efficacy of EGL2 in controlling infections by *E. amylovora* and *X. fastidiosa* was evaluated in potted pear and almond plants, respectively, and under greenhouse conditions.

#### 2.6.1 *Erwinia amylovora* assays

Self-rooted pear plants cv. Conference (3-year-old) were used. Plants were pruned to leave 3-4 shoots per plant, forced to sprout in the greenhouse and used when shoots contained 5 to 6 young leaves per shoot. Plants were fertilized once a week with a 200 ppm of water soluble NPK solution (20:10:20). Disease was evaluated in leaves of plants that have been sprayed until drop-off with aqueous solution of EGL2 at 20 and 40 µl/ml (10 ml per plant). Streptomycin (0.10 mg/ml) was used as a reference control product, and water-sprayed plants were used as non-treated controls (NTC). Before treating the plants with different EGL2 doses, a double transverse incision (*ca*. 1mm) was made perpendicular to the midrib of the three youngest fully expanded leaves (most susceptible to infection) of each shoot. After 24 h, treated plants were inoculated with the pathogen by delivering 10 µl of bacterial suspension (at 5x10^7^ CFU/ml) to the center of the two incisions previously made in the midrib. Plants were incubated in the controlled environment greenhouse at 23 ± 2 °C and a photoperiod of for 16 h of light and 8 h dark and 60% relative humidity. The experimental design consisted of three biological replicates of three plants per each treatment and pathogen. Two independent experiments were performed.

After incubation, disease symptoms were allowing to develop and the intensity of the infections was scored 8 days after pathogen inoculation, using a severity index ranging from 0 to a maximum of 4 (0, no symptoms; 1, localized necrosis around the wound; 2: complete necrosis of the midrib; 3, progression of the necrosis across the petiole and 4, progression of necrosis towards shoot). In every plant, each of 3 leaves belonging to each shoot was rated according to the index, and it was used to calculate a disease severity index per plant according to the formula:


$$S=\sum_{i=1}^{n}\frac{Ii}{\left(n.4\right)}x100$$

where S is the severity of the infections per plant, I_i_ is the severity index for each leaf, n is the number of leaves measured, which is multiplied by the maximum severity index (i.e. 4). Then, the mean of the three plants for each biological replicate was used for the statistical analysis.

#### 2.6.2 *Xylella fastidiosa* assays

The effect of EGL2 on disease severity caused by *X. fastidiosa* subsp. *fastidiosa* 5387.2 and subsp. *multiplex* 5901.2 in inoculated almond plants was assessed. Almond plants were inoculated as detailed in Baró et al. (2021). Briefly, three inoculations of 10 µl each (30 µl of inoculum per plant, total inoculum of 3x10^6^ CFU) were applied at the same side of the stem in a section of 3 cm at around 15 cm above the substrate level, with a high-precision microinjector (NanoJet; Chemyx, Stafford, TX) provided with a Hamilton 250-µl syringae with a thin needle with a beveled tip (Bonaduz, Switzerland).

In a first experiment, 20 or 40 µl/ml of EGL2 was applied preventively by endotherapy following the protocol described in Baró et al. (2021) for pathogen inoculation. In a second experiment, four independent strategies of 60 µl/ml EGL2 treatment were explored and consisted of (1) preventive application by endotherapy 1 day before the inoculation (1dbi) of *X. fastidiosa* (2) combination of preventive (1 dbi) and curative application by endotherapy 7- and 43-days post-inoculation (dpi) (3) preventive application (1 dbi) by soil drench, and (4) combination of preventive (1 dbi) and curative application by soil drench 7 and 43 dpi.

At the end of the period of treatment the plants that have been treated by endotherapy using the preventive strategy received a total of 1.8 µl/plant, whereas the plants treated by endotherapy using the combined strategy (preventive and curative) received a total of 5.4 µl/plant (corresponding to three applications of 1.8 µl/plant, one preventive application and two additional curative applications). The experimental design consisted of nine plants per each treatment, and two independent experiments were performed. Symptoms were evaluated according to the severity scale previously described (Baró et al., 2021). For both pathosystems, data set was subjected to analysis of variance (one-way ANOVA) to determine if there were significant differences between treatments in bacterial disease control. Efficacy of each treatment was calculated based on the severity of the treatment in relation to severity observed in plants NTC group, according to the formula:


$$E\;\left(\%\right)=\frac{SNTC-ST}{SNTC}x\;100$$

where *E* is the efficacy of the treatment (in percentage), *SNTC* is the severity observed in the plants of the NTC group and *ST* is the severity observed in the treatment group.

Infected plants were cultivated in a Biosafety level II+ quarantine greenhouse authorized by the Plant Health Services, according to EPPO recommended containment conditions (EPPO, 2006), taking into consideration *X. fastidiosa* quarantine status in the EU (EFSA PLH Panel et al., 2018).

### 2.7 Quantitative PCR

The levels of *X. fastidiosa* in inoculated plants were analyzed by quantitative PCR as described by Baró et al. (2021). To determine the movement and growth of *X. fastidiosa*, 16 cm of shoot material consisting of two sampling zones located above (Upwards zone 1, U1; Upwards zone 2, U2; 8 cm each zone) and below the inoculation point (Zone, D; 8 cm). *X. fastidiosa* levels were analyzed in each of nine plants per treatment and in each zone. Briefly, after removing the bark from each zone and processing the plant material, DNA extraction and purification was performed using the GeneJET Genomic DNA purification kit (Thermo Fisher Scientific, USA) following the manufacturer’s instructions. The number of total *X. fastidiosa* cells in the xylem tissue was quantified using a quantitative PCR and expressed as CFU/g, by interpolating the C_T_ values of each sample in the standard curve, C_T_ values *vs.* CFU, described previously (Baró et al., 2020b).

### 2.8 Transcriptomic analysis of almond plants challenged with EGL2

The effect of EGL2 in almond plant defense response was evaluated using two preventive application approaches, endotherapy or soil drenching, as detailed in section 2.6 (*X. fastidiosa* section, preventive application). Control plants were treated with distilled water. For RT-qPCR analyses, treated leaves were collected 24 h after treatment and immediately frozen in liquid nitrogen for subsequent RNA extraction. For total RNA extraction, the plant material was ground to a fine powder in liquid nitrogen with the Tissuelyzer II system (Qiagen) and total RNA was extracted using TriZol^®^ (Invitrogen, Life Technologies) followed by DNAse treatment (Ambion^®^ Turbo DNA-free™, Life Technologies) to remove any contaminant DNA, as described in Montesinos et al. (2021). RNA samples of 3 plants were pooled in the same Eppendorf tube, and three biological replicates per treatment were analyzed (9 plants/treatment, 3 tubes per treatment). cDNA was synthesized from RNA samples using High-Capacity cDNA Reverse Transcription Kits (Thermo Fisher Scientific Inc.) and was assayed for quantify the expression levels of eight *Prunu*s genes related to plant defense and previously described in Ruiz et al. (2017); Tong et al. (2009); Foix et al. (2021). These genes codify a basic 7S globulin-like protein, glutamate receptor, a WRKY transcription factor, G-type lectin S-receptor-like serine/threonine-protein kinase, a pathogenesis-related transcriptional activator PTI5, a PR9 (a peroxidase 44), a RING-H2 finger protein and a pathogenesis-related protein 4 (PR4). Primers are detailed in **Supplementary Table 3**.

Quantitative Real Time-PCR was carried out in a fluorometric thermal cycler (qPCR Quant Studio 5, Applied Biosystems) using the Mix SYBR^®^Green PCR Master Mix (Applied Biosystems) as described in Badosa et al. (2017). The total reaction volume was 20 µl containing 1x Sybr Green Master Mix (Applied Biosystems), the appropriate concentration of primers (Sigma) and 2 µL of RT reaction (cDNA). The reaction conditions were as follows: (1) initial denaturation step (10 min at 95°C); (2) amplification and quantification (50 cycles of 15 s at 95°C and 1 min at 60°C); and a final melting program (60-95°C with a heating rate of 0.5°C/s) as described in Badosa and co-workers (2017). Reactions were carried out in duplicate in 96-well plates. Controls from no cDNA template were included as negative controls. The relative quantification of each individual gene expression was performed using the 2^-ΔΔCt^ method (Livak and Schmittgen, 2001) and the REST2009 Software (Pfaffl et al., 2002). Relative expression values of each plant defense were calculated normalizing against the *UBQ-*gene as an internal control.

### 2.9 Data analysis

To test the effect of EGL2 on the population levels of *X. fastidiosa* on almond plants and disease severity in almond and pear plants, an analysis of variance (one-way ANOVA) was performed. Means were separated according to the Tukey’s test at a *P* value of ≤ 0.05. The statistical significance of the gene expression data was determined using the REST2009 Software (Pfaffl et al., 2002).

## 3 Results

### 3.1 Antibacterial activity

Antibacterial activity of EGL2 against the strains of plant pathogenic bacteria is shown in **Table 1**. All bacteria were susceptible to EGL2, but there was a differential susceptibility among strains, being the three subspecies of *X. fastidiosa* and *Xanthomonas fragariae* the most sensitive and *E. amylovora* the most tolerant. Specifically, EGL2 showed a MIC value of <5 µl/ml in *X. fastidiosa* and *X. fragariae*, and between 60 and 120 µl/ml in *E. amylovora*.

**Table 1:** Minimal inhibitory concentration (MIC) and bactericidal concentration (MBC) of Eucalyptus essential oil against plant pathogenic bacteria.

		MBC (µl/ml)	
Plant pathogenic bacteria	MIC (µl/ml)	30 min	2 h
*Erwinia amylovora*	60-120	< 10	< 10
*Xanthomonas arboricola* pv. pruni	10-30	> 40	7.5 - 15
*Pseudomonas syringae* pv. actinidiae	10-30	3.75 – 7.5	3.75 – 7.5
*Pseudomonas syringae* pv. tomato	30-60	7.5 - 15	< 7.5
*Pseudomonas syringae* pv. syringae	30-60	-	-
*Xanthomonas fragariae*	< 5	-	-
*Xanthomonas axonopodis* pv. vesicatoria	10-30	> 40	7.5 - 15
*Ralstonia solanacearum*	30-60	-	-
*Xylella fastidiosa* subsp*. fastidiosa*	< 5	< 0.75	< 0.75
*Xylella fastidiosa* subsp*. multiplex*	< 5	< 0.75	< 0.75
*Xylella fastidiosa* subsp*. pauca*	< 5	< 0.75	< 0.75

-, no data available.

**Figure 1:**
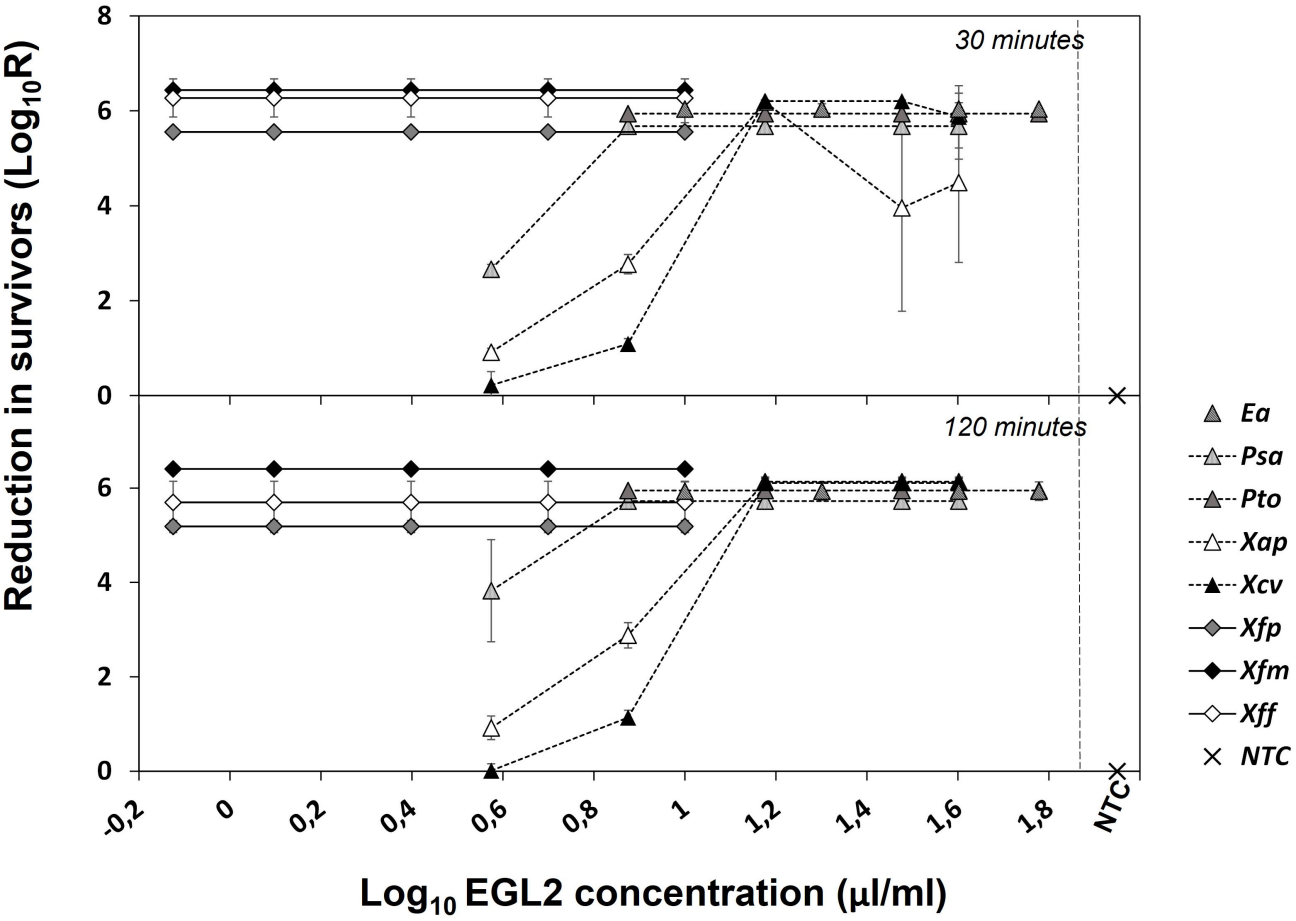
Effect of EGL2 in cell survival of different plant pathogenic bacteria after exposure to 30 or 120 minutes. *Ea*, *Erwinia amylovora; Psa, Pseudomonas syringae* pv. actinidiae; *Pto, Pseudomonas syringae* pv. tomato; *Xap, Xanthomonas arboricola* pv. pruni; *Xcv*, *Xanthomonas campestris* pv. vesicatoria; *Xfp*, *Xylella fastidiosa* subsp. *pauca*; *Xfm, Xylella fastidiosa* subsp. *multiplex; Xff, Xylella fastidiosa* subsp. *fastidiosa.* Non-treated controls (NTC) were included. Values are the means of three replicates, and error bars represent the standard deviation of the mean. Most bacteria were highly susceptible to EGL2 with high reduction in survivors even at the lowest concentration of 0.75 µl/ml. *Psa*, *Xap* and *Xcv* were the less susceptible.

The bactericidal activity (*killing assay*) was determined against 8 plant pathogenic bacteria selected based on the MIC values (**Figure 1**). Reduction of bacterial populations (survival) varied according to the strain, EGL2 concentration and the exposure time. EGL2 exhibited rapid and potent bactericidal effect and, after 30 min of exposure, a maximum of > 5.7 to 6.0 log reduction (N_0_/N) in survival of *E. amylovora*, *P. syringae* pv. actinidiae, *P. syringae* pv. tomato, and *X. campestris* pv. vesicatoria was observed after incubation at 10, 7.5 and 15 µl/ml, respectively. EGL2 showed also bactericidal activity against *X. arboricola* pv. pruni, although we observed a lower survival reduction of 4.5 logs. Interestingly, a reduction of 5.6 to 6.4 logs in any of the three subspecies of *X. fastidiosa* was observed at 0.75 µl/ml. After increasing the exposure time to 120 min, the reduction of *X. arboricola* pv. pruni survival increased to 6.1 logs after incubation at 15 µl/ml. MBC values are detailed in **Table 1**.

### 3.2 Lytic effect of EGL2 on *X. fastidiosa* cells

**Figure 2:**
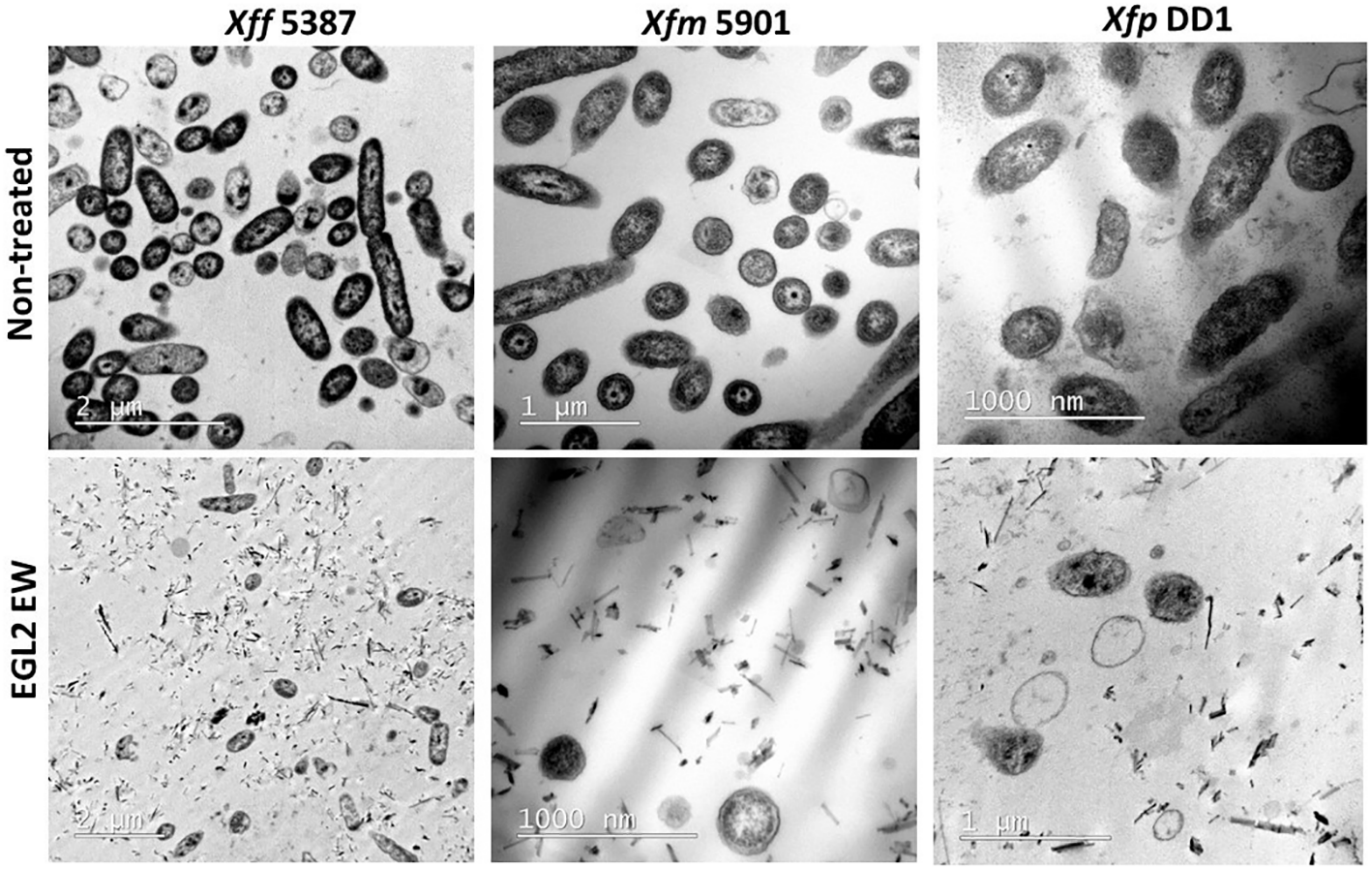
TEM microscopy of samples of of *X. fastidiosa* subsp. *fastidiosa* IVIA 5387.2, subsp. *multiplex* IVIA 5901.2 and subsp. *pauca* De Donno after exposure to EGL2 at 6 µl/ml for 120 min. Notice that very few cells remain intact after the treatment, and extensive lysis and debris material is observed (lower panels).

TEM imaging revealed a potent lytic activity of EGL2 (at 6 µl/ml) against the three *X. fastidiosa* subspecies (**Figure 2**). The vast majority of *X. fastidiosa* cells lost the structural integrity and morphology of the membrane and the cell wall, compared to the non-treated control cells that maintained the cell wall integrity after 2 h exposure.

### 3.3 Effect of EGL2 treatment on bacterial infections in potted plants

#### 3.3.1 *Erwinia amylovora* assays

**Figure 3:**
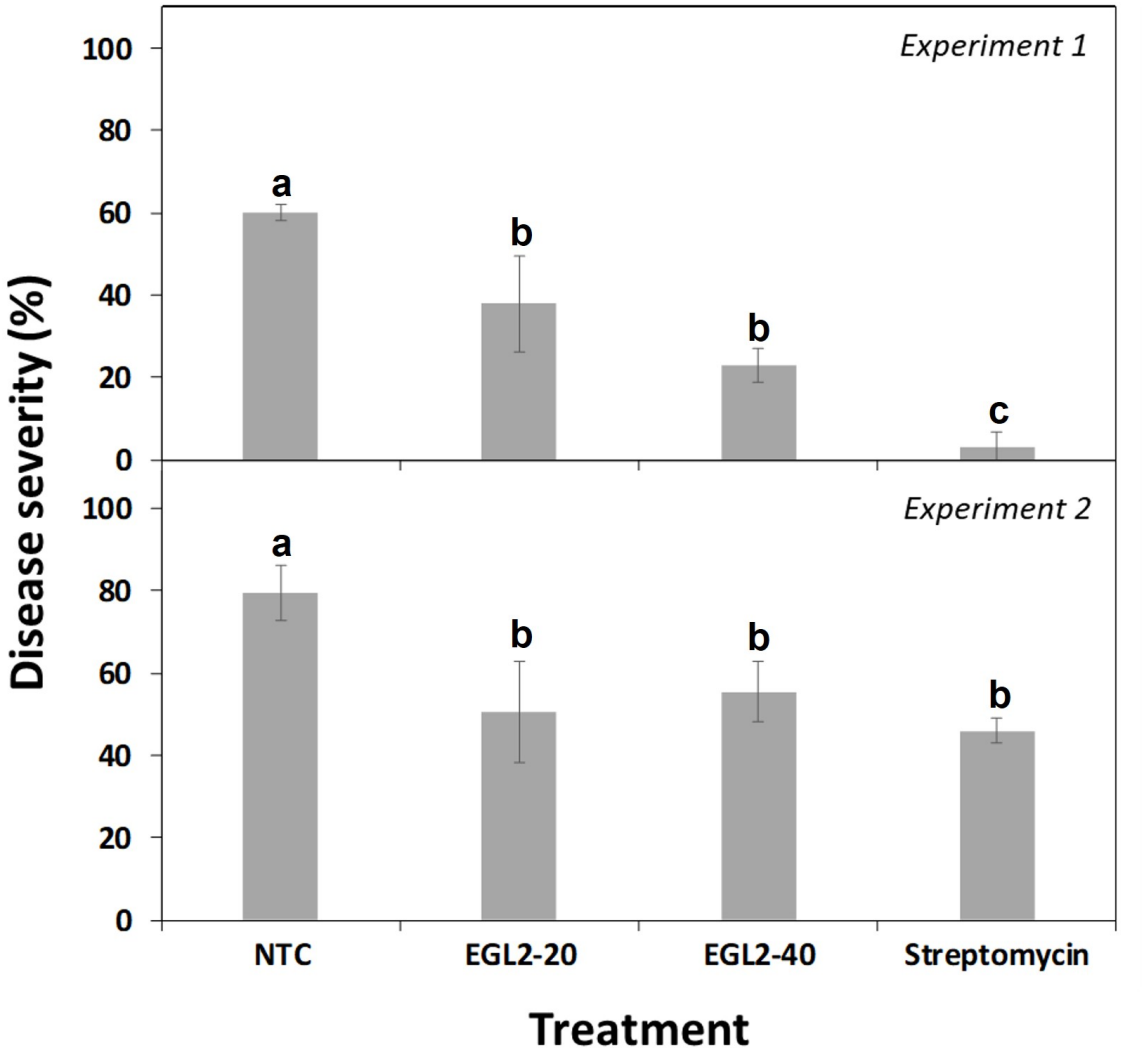
Effect of spray application of EGL2 on infection severity by *E. amylovora* on pear plant leaves. Two independent assays were performed, and EGL2 was applied by spraying 24 h before pathogen inoculation. Disease severity was evaluated on pear plants after 8 days from pathogen inoculation (10^7^ CFU/ml). Values correspond to the mean disease severity of three replicates of three plants per treatment. Standard errors are indicated on bars. The effect of treatments was significant according to ANOVA (p<0.001 for experiment 1 and p=0.002 for experiment 2). Different letters between treatments indicate significant differences between disease severities according to Tukey’s test (P ≤ 0.05).

The preventive spray of EGL2 on pear plants was effective in reducing severity of infections caused by *E. amylovora* (**Figure 3**). Although a significant difference between the two experiments conducted was observed (F=102.4; P<0.0001), the reduction effect was consistent (F=50.5; P<0.0001). More in detail, after treatment with 20 µl/ml, disease severity was 37.9% for experiment 1 and 50.6% for experiment 2 (37.0 and 36,7% efficacy, respectively), and after treatment with 40 µl/ml it was of 23.0% and 55.5% (39.4 and 30.2% efficacy), compared to non-treated controls (60.2% in experiment 1 and 79.5% in experiment 2). No significant differences were observed among EGL2 doses. In the second experiment no significant differences were observed between streptomycin and EGL2 treatment. No phytotoxic effects were observed on the treated pear plants.

#### 3.3.2 *Xylella fastidiosa* assays

**Figure 4:**
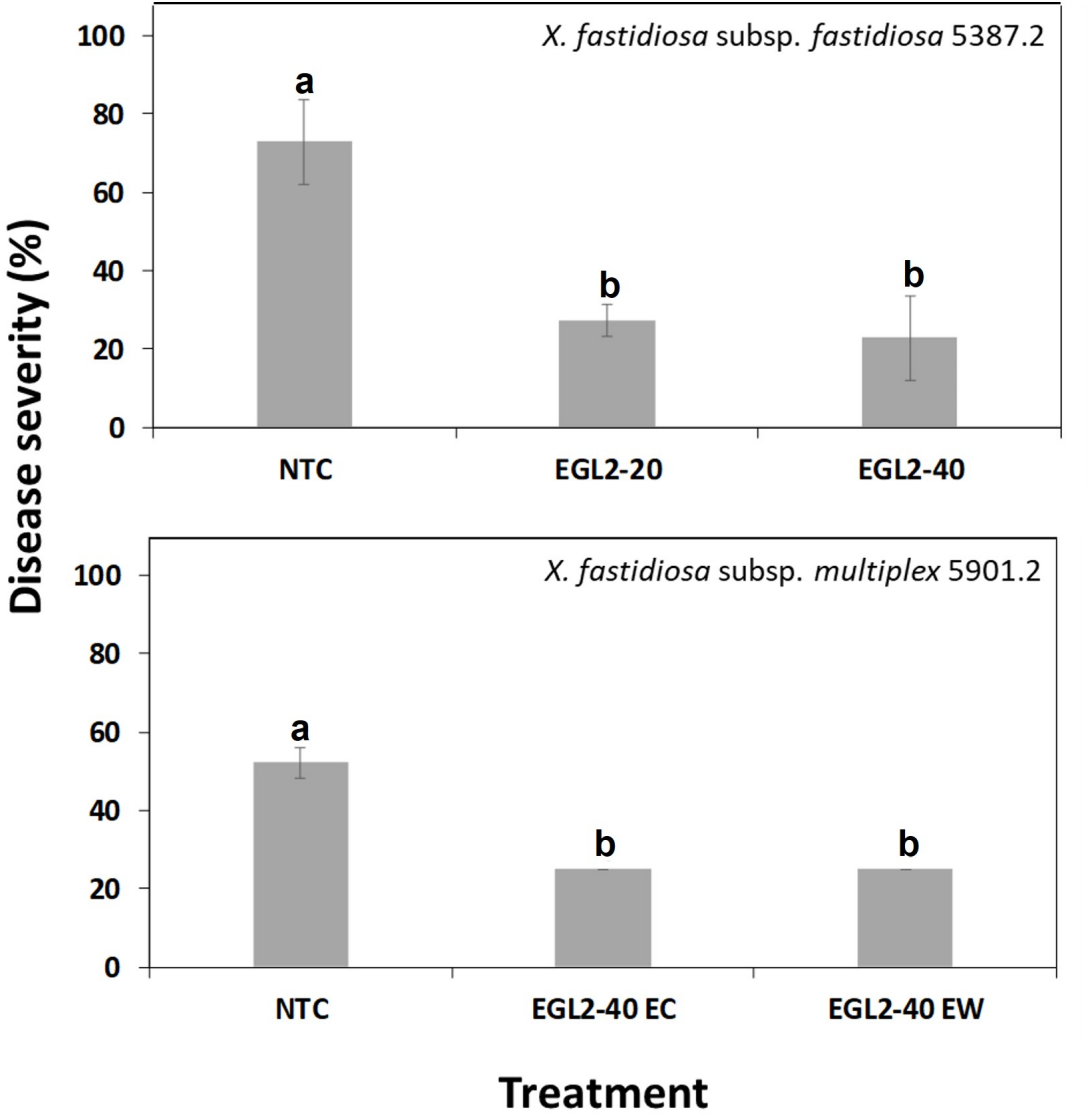
Disease severity of ALS in almond plants treated with EGL2 by endotherapy and infected by *X. fastidiosa* subsp. *fastidiosa* IVIA 5387.2 or *X. fastidiosa* subsp. *multiplex* IVIA 5901.2. Values are the means of 3 replicates of 3 plants, and error bars represent the standard deviation of the mean. The effect of treatments was significant according to ANOVA (p=0.006 for experiment 1 and p<0.001 for experiment 2). Different letters between treatments indicate significant differences between disease severities according to Tukey’s test (P ≤ 0.05).

**Figure 5:**
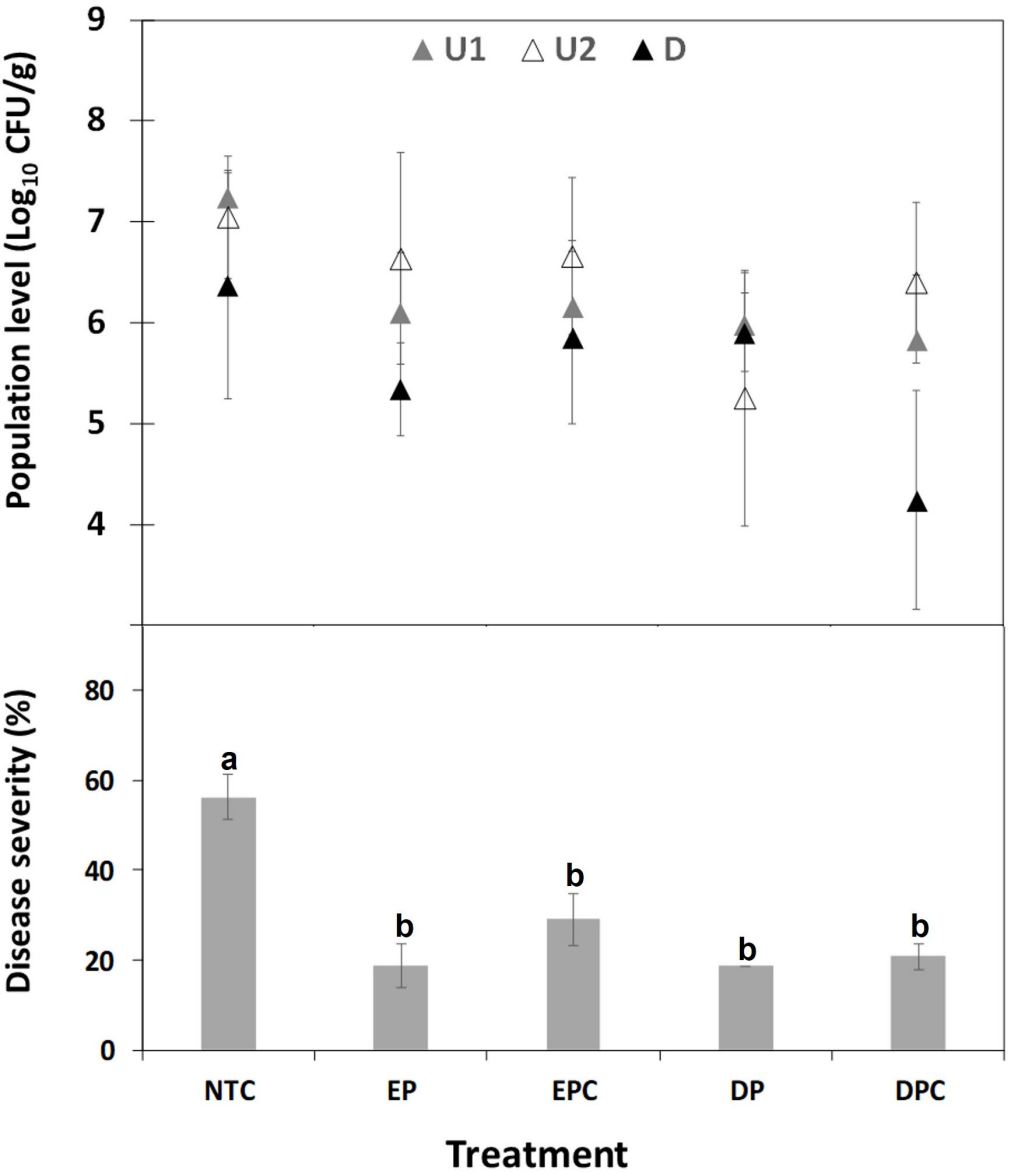
Effect of EGL2 treatment strategies on *X. fastidiosa* subsp. *fastidiosa* IVIA 5387.2 population levels in almond plants after 70 days post inoculation. Four independent strategies of EGL2 treatment (60 µl/ml) were used and consisted of: (1) preventive application by endotherapy 1 day before the pathogen inoculation (1dbi, EP); (2) combination of preventive (1 dbi) and curative application by endotherapy 7 and 43 days post-inoculation (dpi, EPC); (3) preventive application (1 dbi, DP) by soil drench, and (4) combination of preventive (1 dbi) and curative application by soil drench 7 and 43 dpi (DPC). Sampled zones are also indicated as upwards zones (U1 and U2) and downwards zone (D), in relation to the point of inoculation and product injection. Values are the means of 6 plants, and error bars represent the standard deviation of the mean. The effect of treatments was significant according to ANOVA (p<0.0001). Different letters between treatments indicate significant differences between disease severities according to Tukey’s test (P ≤ 0.05).

Plants treated with water showed the typical symptoms of ALS disease, earlier than EGL2 treated plants. Consistently, EGL2 decreased the intensity of infections compared to NTC. In the first experiment (**Figure 4**), the preventive application of EGL2 by endotherapy controlled infections caused by both *X. fastidiosa* subsp. *fastidiosa* as well as *X. fastidiosa* subsp. *multiplex* in almond plants, with efficacies of 68.6% and 52%, respectively. In the second experiment (**Figure 5**), different application strategies were studied. EGL2 preventive application, either by endotherapy or soil drenching resulted in efficacies of 66.7%. The combined strategy (combination of preventive and curative application) using soil drenching or endotherapy allowed an efficacy of 63% and 48.1%, respectively.

Throughout the second experiment, the levels of *X. fastidiosa* in almond plants were quantified after the treatment with EGL2, and compared to non-treated control plants after 30 and 70 dpi. At 30 dpi, no significant differences were observed between treatments. The levels in different plant zones (U1, U2, D) after 70 dpi are shown in **Figure 5** and **Supplementary Figure 1**. At 70 dpi, after EGL2 application, a consistent decrease in *X. fastidiosa* levels was observed in U1, compared to non-treated control plants (reduction of 1.1 to 1.4 logs) by soil drench or endotherapy, in preventive or combined strategies (preventive and curative) (**Figure 5** and **Supplementary Figure 1**). No significant differences were observed between strategies used. The combination of preventive and curative application by endotherapy reduced *X. fastidiosa* cells in U2 zone, with a decrease of 1.8 logs. The combined treatment using soil drenching was the optimal one in reducing *X. fastidiosa* cells in the basal zone with a decrease of 2.1 logs.

No phytotoxic effects were observed on the treated almond plants.

### 3.4 Effect of EGL2 treatment on the expression of defense-related genes in almond

The almond response to EGL2 was determined in almond plants preventively treated by soil drenching or endotherapy, and using a selection of genes related to plant defenses (Foix et al., 2021). The expression of 8 plant defense-related genes was analyzed after 24 h, and transcriptomic changes were only observed when plants were treated by endotherapy (**Table 2**). Specifically, after endotherapy, transcript levels increased for the genes *7S globulin-like, glutamate receptor, G-type lectin S-receptor-like serine/threonine-protein kinase, pathogenesis-related transcriptional activator PTI5, PR9, RING-H2 finger* and *pathogenesis-related protein 4*, in comparison to non-treated control plants.

**Table 2:** Expression of genes related to plant defense response in *Prunus dulcis* after Eucalyptus essential oil treatments, applied by two different strategies.

	EGL2 application strategy	
Gene	Soil drenching	Endotherapy
*7S globulin*	0.8	**3.1**
*GLR 2.7*	1.1	**12.9**
*WRKY 33*	0.9	0.0
*SRL*	1.0	**1.5**
*PTI5*	0.9	**3.7**
*PR9*	0.6	**1.9**
*RING H2*	1.0	**2.9**
*PR4*	1.3	1.0

Values in bold correspond to significant overexpression. The statistical significance of the gene expression data was determined using the REST2009 Software (Pfaffl et al., 2002).

## 4 Discussion

In the present study we investigated the antibacterial properties of a Eucalyptus EO, the ELG2 formulation, against representative plant pathogenic bacteria, and its effect to enhance defense priming responses in a plant host, that finally improved infection control in two bacterial pathosystems.

EGL2 showed a potent *in vitro* bactericidal activity against *E. amylovora*, *P. syringae* pv. syringae, *P. syringae* pv. tomato, *X. campestris* pv. vesicatoria, *X. arboricola* pv. pruni, *X. fastidiosa* subsp. *fastidiosa*, *X. fastidiosa* subsp. *pauca* and *X. fastidiosa* subsp. *multiplex*. The bactericidal activity was dependent on the EO concentration, bacterial strain, and the exposure time, and the most susceptible bacteria were the three subspecies of *X. fastidiosa* with an MBC of 0.75 µl/ml (750 ppm). Globally, these results agree with reports of other EO showing antibacterial activity against phytopathogenic bacteria such as *X. fastidiosa*, *Agrobacterium tumefaciens* or *Clavibacter michiganensis* (Alonso-Gato et al., 2021). However, it is difficult to compare our data with other studies because of the differences in methods, units of concentration, and bacterial strains used, and in most cases focused food preservation and the medical field. Also, the chemical composition and consequently the antimicrobial activity of EOs, even from a same plant species, can differ due to the method of extraction, its origin, plant organ, geographical location, or climatic conditions (Razzouk et al., 2022). In addition, there are only few reports on Eucalyptus EO (Pandey et al., 2017; Raveau et al., 2020).

Despite these limitations, some comparisons can be done. For example, it has been reported that the MIC of an essential oil of *Eucalyptus globulus* fruits ranged from 3-4 mg/ml (3000-4000 ppm) against *Pseudomonas aeruginosa*, *Staphylococcus aureus*, *Bacillus subtilis* or *Escherichia coli*, while the MBC varied between 3.6 to 9.0 mg/ml (3600 to 9000 ppm) (Bakkali et al., 2008; Said et al., 2016). Globulol, the main component of *E. globulus* fruit petroleum ether fraction of the ethanol crude extract, showed IC_50_ values on *Xanthomonas vesicatoria* and *Bacillus subtilis* of 158.0 µg/ml and 737.2 µg/ml, respectively (Tan et al., 2008). EL-Hefny et al. (2017) find, by disc diffusion susceptibility test, that MICs of extracts of *Eucalyptus camaldulensis* ranged from 16 to 500 ppm against *Pectobacterium carotovorum*, *Ralstonia solanacearum*, *Dickeya* spp., and *Agrobacterium tumefaciens*. Similar results were reported with EO extracts from different plants like *Rosa damascene* or *Thymbra spicata* against *Erwinia amylovora*, (MBC 1.4 mg/ml (1400 ppm) and 0.5 mg/ml (500 ppm), respectively), *Russowia sogdiana* against *A. tumefaciens* (MIC 0.2-to 0.8 mg/ml (200-800 ppm) and *Cleistocalyx peraculatus* against *X. campestris* pv. vesicatoria (MBC 62.5 to 250 µg/ml (62.5 to 250 ppm) (Basim and Basim, 2004; Tan et al., 2007; Bajpai et al., 2010). In the case of *Xylella fastidiosa* 9a5c, Brentini et al. (2018) determined for a Eucalyptus oil a MIC of 1000 µg/ml, much higher than we observed after exposure to ELG2 of *X. fastidiosa* subsp. *fastidiosa* 5387.2, *X. fastidiosa* subsp. *multiplex* 5901.2 or *X. fastidiosa* subsp. *pauca* De Donno (MICs <5 μl/ml).

The bactericidal activity of EGL2 against the three subspecies of *X. fastidiosa* was based on a lytic effect, according to TEM ultrastructural imaging. This observation agrees with the fact that terpenes, the main component of essential oils, can disrupt the bacterial cell membrane structure by releasing the lipopolysaccharides and resulting in bacterial cell permeabilization (Sharifi-Rad et al., 2017).

In the present study, apart from the bactericidal effect of EGL2, induced disease tolerance has been associated to overexpression of plant defense genes. To the best of our knowledge, there are no reports on the stimulation of plant defense by Eucalyptus EO. However, our findings agree with the fact that many EOs are involved in host defense mechanisms against plant pathogens, resulting in reduction of disease development (Batish et al., 2008). For example, the foliar application of ethanolic extracts from Giant knotweed induced SA-dependent defense responses in cucumber plants, and reduce powdery mildew severity (Margaritopoulou et al., 2020). Likewise, lavender EO induced overexpression of genes related to SA and ethylene/jasmonic acid pathways in sorghum plants (Rashad et al., 2022). More specifically, we have identified that treatment of almond by endotherapy with EGL2 overexpressed genes coding a vicilin protein (also known as 7S globulin), a glutamate receptor, a G-type lectin S-receptor-like serine/threonine protein kinase, a pathogenesis-related transcriptional activator PTI5, a PR9 (Per44), a RING-H2 finger and a pathogenesis-related protein 4 (PR4).

Glutamate receptors, G-type lectin S-receptor-like serine/threonine protein kinases (LecRLKs) and pathogenesis-related transcriptional activators PTI5 are described as positive regulators of PAMP triggered immunity (PTI) (Luo et al., 2017). In addition, glutamate receptor also responds to an attack from pathogens (and wounding) by mediating PTI by acting as Ca^2+^ channels (Forde and Roberts, 2014). LecRLK has been also described as a positive regulator of plant tolerance to salt stress (Sun et al., 2013) and plays crucial roles in plant development and responses to biotic and abiotic stresses (Han et al., 2021). In case of *LecRLK* overexpression, it has been reported that pathogen infection due to *P. syringae* DC3000 activates not only the transcription of *LecRLK* in *Arabidopsis*, but also plants overexpressing *LecRLK* show not only resistant phenotype to *P. syringae* DC3000 but also reactive oxygen species (ROS)-production and SA accumulation (Luo et al., 2017). In relation to *PTI5* it is known that belongs to the ethylene-response factor (*ERF*) family and binds to promoters of many *pathogenesis-related* (*PR*) genes (Yong-Qiang et al., 2002), and so overexpression of *PTI5* in tomato enhanced protection to *P. syringae* pv. tomato (He et al., 2001) while overexpression in *Arabidopsis* activated the expression of SA-regulated genes (*PR1* and *PR2* genes) (Gu et al., 2002).

Additionally, the pathogenesis-related proteins PR4 and PR9, and vicilin, also seem to contribute to highest tolerance to *X. fastidiosa* infection. Specifically, the induction of *PR9 (Per44)* is related with plant defense against pathogen attack and environmental stresses, being a key component of ROS production during plant defense responses (Soylu et al., 2005). Moreover, PR9 plays a key role in lignin biosynthesis and biodegradation, contributing to strengthening plant cell walls by catalyzing lignin deposition (Taheri and Tarighi, 2012). Likewise, PR4 is crucial against a fungal attack, but also increased its synthesis by other biotic factors such as bacteria, virus, insects as well as abiotic stresses, SA, or ethylene (Sharma et al., 2011; Grove, 2012), and vicilin is a multifunctional protein related to stress responses, antibacterial activity, and hormone receptor-like activity, and in some plants are described as a precursor of antimicrobial peptides (Hirano, 2021).

We have demonstrated here that the preventive application of EGL2 applied by spraying to pear plants reduced *E. amylovora* infections with an efficacy close to 40%. Since the first report on the antibacterial activity of the active substances derived from EO (i.e., the terpenoids geraniol and citronellol) against *E. amylovora* (Scortichini and Rossi, 1991), several studies have been conducted using detached organs (Măruţescu et al., 2009; Akhlaghi et al., 2020), but none demonstrating the effectiveness of EO treatment in fire blight control using *in planta* assays. In addition, EGL2 applied to almond plants by different strategies (endotherapy and soil drenching, preventive, curative or a combination of both), reduced severity of infections caused by *X. fastidiosa*, under greenhouse conditions, with efficacies varying from 50 to 70%. The reduction in ALS symptoms was associated to a reduction in levels of *X. fastidiosa* in treated plants, although pathogen was not eliminated. Our results of efficacy of Eucalyptus EO, agree with the field reports of different compounds such as a biocomplex of zinc, copper and citric acid, N-acetylcysteine, fosetyl-aluminum, bioactive detergent from plants, diffusible signal factor, as well as endophytic microorganisms, avirulent/weakly virulent *X. fastidiosa* strains or bacteriophages, that in some cases reduced symptoms in infected plants, but were not able to eliminate *X. fastidiosa* from diseased plants (EFSA PLH Panel et al., 2019; Morelli et al., 2021).

In conclusion, our study demonstrates that a formulation of Eucalyptus EO has a strong bactericidal effect and can protect almond plants from *X. fastidiosa* and pear plants from *E. amylovora* infections, on plants under greenhouse conditions. The EGL2 acts by a dual mechanism, directly against the target plant pathogenic bacteria and indirectly by eliciting defense responses in the host plant. This bifunctional mechanism of action and the fact that can be applied using different methods (irrigation, endotherapy, spray) demonstrates its potential for controlling bacterial diseases of plants.

## Data availability statement

The original contributions presented in the study are included in the article/**Supplementary Materials**, further inquiries can be directed to the corresponding author/s.

## Author contributions

EM obtained the financial support. EM and LM designed research and wrote the paper. LM, AB, and BG conducted, performed the experiments, and analyzed de data. All authors contributed to the article and approved the submitted version.

## Supplementary material

Supplementary Figure 1 Effect of EGL2 treatment strategies on *X. fastidiosa* subsp. *fastidiosa* IVIA 5387.2 population levels in almond plants after 70 days post inoculation. Four independent strategies of EGL2 treatment (60 µl/ml) were used and consisted of: (1) preventive application by endotherapy 1 day before the pathogen inoculation (1dbi, EP); (2) combination of preventive (1 dbi) and curative application by endotherapy 7 and 43 days post-inoculation (dpi, EPC); (3) preventive application (1 dbi, DP) by soil drench, and (4) combination of preventive (1 dbi) and curative application by soil drench 7 and 43 dpi (DPC). Sampled zones are also indicated as upwards zones (U1 and U2) and downwards zone (D), in relation to the point of inoculation and product injection. Values are the means of 6 plants, and error bars represent the standard deviation of the mean. Different letters between zones indicate significant differences between population levels of *X. fastidiosa* according to Tukey’s test (*P* ≤ 0.05).
