# Supplementary figures and images for: Bactericidal and plant defense elicitation activities of Eucalyptus oil decrease the severity of infections by Xylella fastidiosa on almond plants
Source: Front Plant Sci. 2023 Mar 15;14:1122218. doi: 10.3389/fpls.2023.1122218 (PMC10050747; doi:10.3389/fpls.2023.1122218)

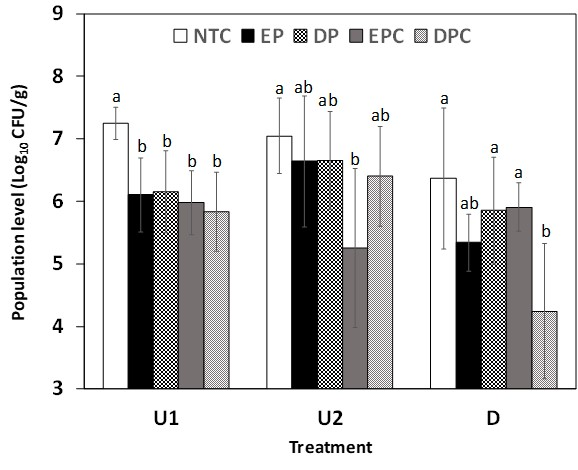

Supplement: Supplementary Figure 1 — Effect of EGL2 treatment strategies on X. fastidiosa subsp. fastidiosa IVIA 5387.2 population levels in almond plants after 70 days post inoculation. Four independent strategies of EGL2 treatment (60 µl/ml) were used and consisted of: (1) preventive application by endotherapy 1 day before the pathogen inoculation (1dbi, EP); (2) combination of preventive (1 dbi) and curative application by endotherapy 7 and 43 days post-inoculation (dpi, EPC); (3) preventive application (1 dbi, DP) by soil drench, and (4) combination of preventive (1 dbi) and curative application by soil drench 7 and 43 dpi (DPC). Sampled zones are also indicated as upwards zones (U1 and U2) and downwards zone (D), in relation to the point of inoculation and product injection. Values are the means of 6 plants, and error bars represent the standard deviation of the mean. Different letters between zones indicate significant differences between population levels of X. fastidiosa according to Tukey’s test (P ≤ 0.05). [file Image_1.tif]
