# Supplementary material for: Bactericidal and plant defense elicitation activities of Eucalyptus oil decrease the severity of infections by Xylella fastidiosa on almond plants
Source: Front Plant Sci. 2023 Mar 15;14:1122218. doi: 10.3389/fpls.2023.1122218 (PMC10050747; doi:10.3389/fpls.2023.1122218)
Supplement: Supplementary file 2 [file Table_1.docx]

Supplementary Table 1. Strains of plant pathogenic bacteria used and growth conditions

| **Bacterias fitopatógenas** | **Strain** | **Country** | **Origin** | **Host plant** | **Culture Medium** |
| --- | --- | --- | --- | --- | --- |
| *Erwinia amylovora* | EPS 101 | Spain | UdG | *Pyrus communis* | LB |
| *Xanthomonas arboricola* pv. pruni | CFBP 5563 | France | CFBP | *Prunus persica* | LB |
| *Pseudomonas syringae* pv. actinidiae | IVIA 3700.1.1 | Portugal | IVIA | *Actinidia deliciosa* | LB |
| *Pseudomonas syringae* pv. tomato | DC 3000 | UK | UN | *Solanum lycopersici* | LB |
| *Pseudomonas syringae* pv. syringae | EPS 94 | Spain | UdG | *Pyrus communis* | LB |
| *Xanthomonas fragariae* | 349-9A | Spain | IVIA | *Fragaria vesca* | Medium B |
| *Xanthomonas axonopodis* pv. vesicatoria | IVIA 2133.2 | Spain | IVIA | *Capsicum annuum* | LB |
| *Ralstonia solanacearum* | CECT 125 | Puerto Rico | CECT | *Solanum lycopersici* | LB |
| *Xylella fastidiosa* subsp*. fastidiosa* | IVIA 5387.2 | Spain | IVIA | *Prunus dulcis* | BCYE, PD2 |
| *Xylella fastidiosa* subsp*. multiplex* | IVIA 5901.2 | Spain | IVIA | *Prunus dulcis* | BCYE, PD2 |
| *Xylella fastidiosa* subsp*. pauca* | De Donno | Italy | CNR | *Olea europea* | BCYE, PD2 |

UdG, University of Girona, Spain; CFBP, French collection of plant pathogenic bacteria; UN, University of Navarra, Spain; IVIA, Valencian Institute for Agricultural Research, Spain; CECT, Spanish Type Culture Collection; CNR, Italian National Research Council, Italy.
