# Supplementary material for: Bactericidal and plant defense elicitation activities of Eucalyptus oil decrease the severity of infections by Xylella fastidiosa on almond plants
Source: Front Plant Sci. 2023 Mar 15;14:1122218. doi: 10.3389/fpls.2023.1122218 (PMC10050747; doi:10.3389/fpls.2023.1122218)
Supplement: Supplementary file 3 [file Table_2.docx]

Supplementary Table 2. Chemical composition of Eucalyptus essential oil and percentages of main components

| **Ingredient** | **Concentration (%)** | **CAS No.** | **EC No.** |
| --- | --- | --- | --- |
| 1,8-Cineole | >70 | 470-82-6 | 207-431-5 |
| Limonene | <10 | 5989-27-5 | 227-813-5 |
| α-Terpineol | <10 | 98-55-5 | 202-680-6 |
| α-Pinene | <5 | 80-56-8 | 201-291-9 |
| ϒ-Terpinene | <5 | 99-85-4 | 202-794-6 |
| р-cymene | <5 | 99-87-6 | 202-796-7 |
| Sabinene | ≤2 | 3387-41-5 | 222-212-4 |
| Phellandrene | ≤1.5 | 99-83-2 | 202-792-5 |
| β-Pinene | ≤1.5 | 127-91-3 | 204-872-5 |

CAS No., Chemical Abstract Service number; EC No., European Community number
