# Supplementary material for: Bactericidal and plant defense elicitation activities of Eucalyptus oil decrease the severity of infections by Xylella fastidiosa on almond plants
Source: Front Plant Sci. 2023 Mar 15;14:1122218. doi: 10.3389/fpls.2023.1122218 (PMC10050747; doi:10.3389/fpls.2023.1122218)
Supplement: Supplementary file 4 [file Table_3.docx]

Supplementary Table 3. Genes and primers used for RT-qPCR analysis to quantify their expression levels in *Prunus dulcis* in response to treatment with Eucaliptus essential oil.

| **Gene** | **Primer code** | **Sequence (5'- 3')** | **References** |
| --- | --- | --- | --- |
| *Basic 7S globulin-like* | 7Sglob_for | TGCAGTCTCTCCCCCGATAA | Foix et al., 2021 |
|  | 7Sglob_rev | AGTGGAACCGCAAGTGAAGA |  |
|  |  |  |  |
| *WRKY transcription factor 33* | WRKY33_for | CCGGTGAGGAAACATGTGGA | Foix et al., 2021 |
|  | WRKY33_rev | CCTCACAGCCATGGACACAT |  |
|  |  |  |  |
| *RING-H2 finger protein* | RingH2_for | TAGGGGAGAGTCGGAGGTTG | Foix et al., 2021 |
|  | RingH2_rev | GGGGCACGTTGCATTCATTT |  |
|  |  |  |  |
| *G-type lectin S-receptor-like serine/threonine-protein kinase* | SRL_for | TGGAAGTGGCTGTCAAGGTC | Foix et al., 2021 |
|  | SRL_rev | TTCAATTCGGGGTCGAAGCA |  |
|  |  |  |  |
| *Pathogenesis-related genes transcriptional activator PTI5* | PTI5_for | CAGCCTCCACGCAAAATGAG | Foix et al., 2021 |
|  | PTI5_rev | AGAGGGTTCGAGGCCATTTG |  |
|  |  |  |  |
| *Glutamate receptor 2.7* | GLR2.7_for | CATCATCGAAGTAGGCCCCC | Foix et al., 2021 |
|  | GLR2.7_rev | GGAATGCCCCTTGCAGTTCT |  |
|  |  |  |  |
| *PR9* | Perox44_for | TCCATAACTGGCGGTTTGCT | Foix et al., 2021 |
|  | Perox44_rev | TGCCTCTAGGCGTTTCTTGG |  |
|  |  |  |  |
| *PR4* | PpPR4_for | TTGCGTACTGCGAGTGTCTT | Ruiz et al., 2017 |
|  | PpPR4_rev | CCATCCATATTTGCTGCGCC |  |
|  |  |  |  |
| *UBQ10* | PpUBQ10ref_for | AAGGCTAAGATCCAAGACAAAGAG | Tong et al., 2009 |
|  | PpUBQ10ref_rev | CCACGAAGACGAAGCACTAAG |  |
